# Dataset of subjective human responses and objective metrics related to motion sickness collected and computed on a winter research voyage

**DOI:** 10.1016/j.dib.2025.111598

**Published:** 2025-04-28

**Authors:** Nicole Catherine Taylor, Anriëtte Bekker, Karel Kruger

**Affiliations:** Department of Mechanical and Mechatronic Engineering, Private Bag X1, Matieland 7602, South Africa

**Keywords:** Motion sickness, Human cyber-physical system, Human factors, Ship motion

## Abstract

The SA Agulhas II, a South African (SA) polar supply and research vessel, embarked on a Winter Research Cruise in July of 2022 with 83 passengers aboard. Subjective human responses related to motion sickness were collected from 63 participants through paperbound questionnaires and, in parallel, from 15 participants through a mobile application that is part of a novel human cyber-physical system. Additionally, the human cyber-physical system enabled participant location tracking using near-field communication technology. The motion sickness responses are answers to questions that describe a participant’s incidence of any motion sickness symptoms and vomiting, as well as the severity of associated symptoms experienced. Ship responses were captured through a full-scale measurement system and processed in real time on board, computing motion sickness dose values for each 5-minute consecutive time series of acceleration measurements. The dataset contains the anonymised human responses to motion sickness and location, the computed motion sickness dose values and down-sampled acceleration measurements for Winter Cruise, which is the first dataset available for data acquired and processed through a shipboard human cyber-physical system. The dataset can be used to compare the concomitant methods of human data collection, compute diagnostic seasickness criteria to serve as a guideline for estimating the incidence of motion sickness on a ship during a voyage, correlate the subjective human and ship responses with each other or used to generate further datasets, for example by using the motion sickness dose values to make objective estimations of the state of motion sickness that can be compared with the subjective responses.

Specifications Table

**Table:** 

Subject	Engineering
Specific subject area	Human factors in shipping – The dataset contains subjective human responses to motion sickness and objective measurements pertaining to motion exposure on board a ship.
Data format	Human responses: anonymised, raw and cleaned Ship responses: filtered, analysed
Type of data	Human responses: Comma separated values in text files and column separated Excel spreadsheets Ship responses: Column separated values in text files Position vectors (supporting document): Column separated Excel spreadsheet
Data collection	Human responses are collected digitally via a custom-developed Android mobile application as an interface to a human cyber-physical system, in parallel to feedback recorded in paperbound booklets with daily questionnaires [1] Ship responses are measured by 5 Direct Current (DC) accelerometers installed shipboard as part of a full-scale measurement system that uses a network of mobile data acquisition systems controlled by LMS Turbine Testing software [2,3]
Data source location	On the SA Agulhas II during the Southern Ocean Seasonal Experiment Winter Research Cruise of 2022. The research cruise track goes from the Port of Cape Town, South Africa, to the marginal ice zone in the Southern Ocean [4].
Data accessibility	Repository name: Ship Motion Measurements and Human Responses Captured on the SA Agulhas II - Winter Cruise 2022. Available on SUNScholarData. https://doi.org/10.25413/sun.24331114.
Related research article	N.C. Taylor, A. Bekker, K. Kruger, 2024. The operational development of diagnostic seasickness criteria through a human cyber-physical system. Applied Ergonomics, 119, 104316. https://doi.org/10.1016/j.apergo.2024.104316.

## 1 Value of the data

• Novel human responses relevant to ship designers, operators and stakeholders about real-world feedback related to motion sickness for improving ship design and operation
• Proof of concept for human-centred data acquisition, monitoring and management through a human cyber-physical system deployed during ship operation
• Insight into differences between human response collection methods on a ship through a digital platform and traditional paper-based method
• Potential to develop seasickness criteria for estimating levels of motion sickness on board a ship to predict and manage motion sickness incidence

## 2 Background

**Fig. 1:**
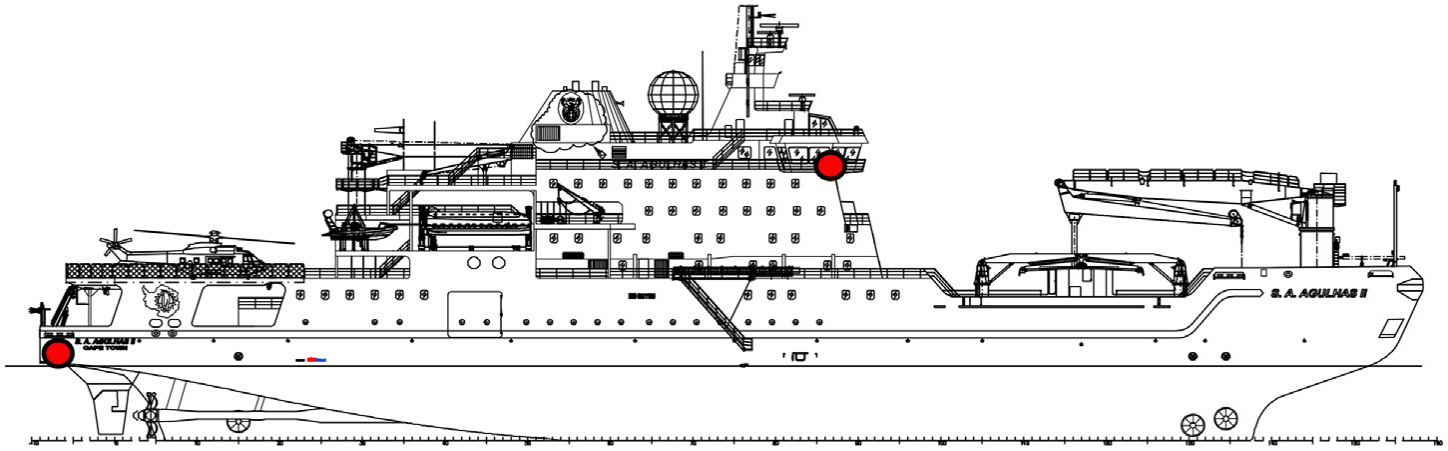
Relevant sensor locations indicated on the SA Agulhas II.

**Fig. 2:**
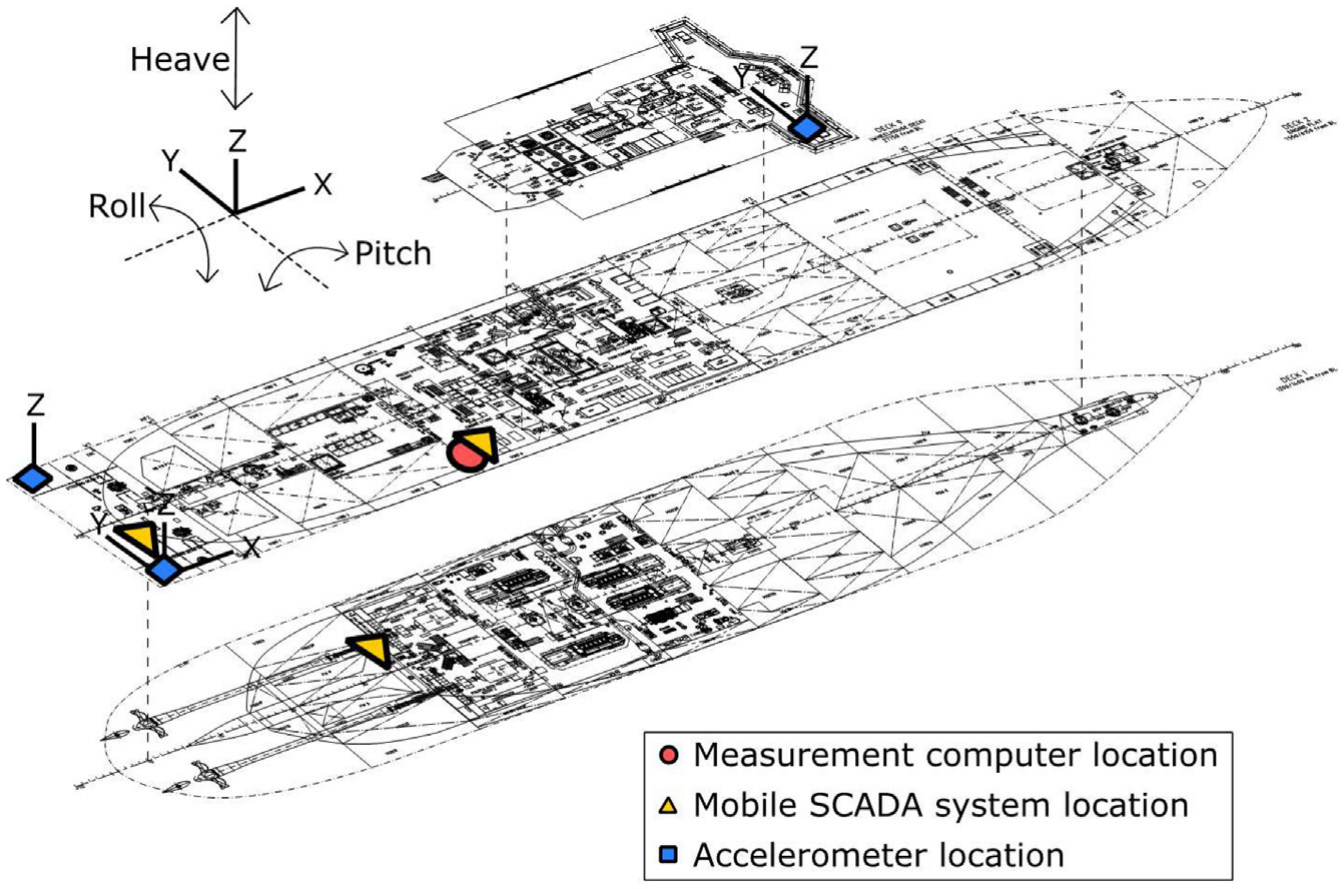
Sensor and measurement equipment positions on the SA Agulhas II.

A consortium is steering a research and measurement campaign that investigates ship and human responses on the SA Agulhas II, South Africa’s (SA’s) polar supply and research vessel [3]. A full-scale sensor network is installed on the SA Agulhas II, with sensor locations relevant to this study shown in Figs. 1 and 2, to study ship responses while navigating through open water and ice conditions to resupply bases in the Southern Ocean and Antarctica. The main ship characteristics are presented in Table 1.

**Table 1:** Main characteristics of the SA Agulhas II [5].

Characteristic	Value
Length overall, *L*_OA_	134 m
Length of waterline, *L*_WL_	126 m
Length between perpendiculars, *L*_PP_	122 m
Beam at waterline, *B*_WL_	22 m
Design draft, *T*_WL_	7.65 m
Depth, *D*	10.6 m
Wetted surface area, *S*	3620 m^2^
Waterplane area, *A*_WP_	2455 m^2^
Displacement, ▽	13687 m^3^
Block coefficient, *C*_B_	0.67
Longitudinal centre of gravity from the aft peak, LCG	57.9 m
Vertical centre of gravity from keel, KG	8.81 m
Metacentric height from keel, GM_t_	2.36 m
Free surface metacentric height corrected, GM_f_	1.63 m
Longitudinal metacentric height, GM_L_	93.5 m
Roll radius of gyration, *k*_44_	0.37*B* m
Pitch radius of gyration, *k*_55_	0.25*L*_PP_ m

The effect of the ship on occupants as a living and workspace for up to 50 crew and 100 passengers is studied as well. Traditionally, feedback from occupants is collected through a paper-based booklet that contains daily questionnaires to report on human factors relating to motion sickness, whole-body vibration and noise on board that are factors known to affect comfort and productivity while seafaring. A human cyber-physical system (HCPS) was developed to immediately digitalise and process, while increasing the frequency of, human responses collected. The HCPS was deployed shipboard for the Winter Research Cruise in July of 2022 with 83 passengers. Of the passengers aboard, 63 voluntarily participated in completing the traditional paper-based booklets daily and, in parallel, 15 used a mobile application to record their responses to motion sickness and track their location on board. This dataset is a collation of the data pertaining to ship and human responses collected during the Winter Research Cruise.

## 3 Data description

The dataset has the file and folder content described in Table 2. The details of the contents for each folder are detailed subsequently.

**Table 2:** File and folder content description.

File/Folder Name	Size	Contents
Readme	261 kB	Detailed description of contents of each folder, including list of files, variables and formats
Winter Cruise Human Responses Collected Through Daily Diaries	64 kB	One .xlsx file containing digitised feedback related to motion sickness collected through paperbound questionnaires
Winter Cruise Human Responses Collected Through the Mariner 4.0 Application	160 kB	30 .txt files containing digitised feedback related to participant location and motion sickness captured through the Mariner 4.0 mobile application
Winter Cruise Acceleration Measurements	1.42 GB	43 folders containing .txt files of acceleration measurements in consecutive 5-min files recorded at specified locations on board captured through DC accelerometers
Winter Cruise Computed Motion Sickness Dose Values	5.26 MB	One .txt file containing the motion sickness dose values at all near-field communication tag locations for the duration of the voyage
Winter Cruise Supporting Documentation	16 kB	One .xlsx file containing position vectors from a specified accelerometer location to near-field communication tag locations vessel wide

### 3.1 Human responses: participant X location or motion sickness – mobile application

Human responses collected through the Mariner 4.0 application installed on participant mobile devices, where ‘X’ is replaced with a numeric identifier to preserve participant anonymity. This includes their location that corresponds to near-field communication (NFC) tags distributed vessel wide or subjective assessments related to their state of motion sickness at any selected time. Motion sickness responses are answers to the questions “Are you motion sick?”, “Did you vomit?” and “What is your illness rating?” (on a scale from 0 to 3, as described in Section 4.1).

### 3.2 Human responses: motion sickness – paperbound questionnaire

Human responses collected through daily diary questionnaire booklets. These are answers to the aforementioned questions, but the assessment is performed once a day at 08h00 (UTC+0h) instead of at any time. Additionally, answers to a once-off questionnaire at the start of the daily diary are included, including demographics of participants related to motion sickness.

### 3.3 Ship responses: acceleration – ship motion data acquisition system

Ship responses captured through accelerometers at positions that enable the computation of motion at any location on board using rigid body dynamics. Each file contains 5 minutes (300 seconds) of acceleration measurements from 5 accelerometers, which is decimated from 2048 Hz to 10 Hz. The accelerometer orientations and locations are presented in Figs. 1 and 2 and in more detail by Taylor *et al.* [1].

### 3.4 Ship responses: motion sickness dose values at NFC tag locations

Motion sickness dose values (MSDVs) were computed for each 5-minute file using z-oriented acceleration translated to each NFC tag location based on methods recommended in ISO 2631-1 (1997) [6]. This created a 5-minute epoch of MSDVs for the full duration of the voyage.

### 3.5 Supporting documentation: position vectors

The position vectors from the accelerometer at location 0 (see the Readme or [1] for more detail) in the Starboard-side Steering Gear Room on Deck 3 to each NFC tag on the SA Agulhas II.

## 4 Experimental design, materials and methods

Human responses were collected during the research voyage from participating passengers through two concomitant methods, including the use of a mobile application that served as an interface to the HCPS and paperbound questionnaires. Ship responses were measured by a full-scale sensor network installed shipboard. All acquired data was validated, primarily using descriptive statistics, to identify potential discrepancies or errors, such as outliers. No data was altered due to veracity screening, as discrepancies or errors were not detected.

### 4.1 Human response acquisition method 1 – mobile application

**Fig. 3:**
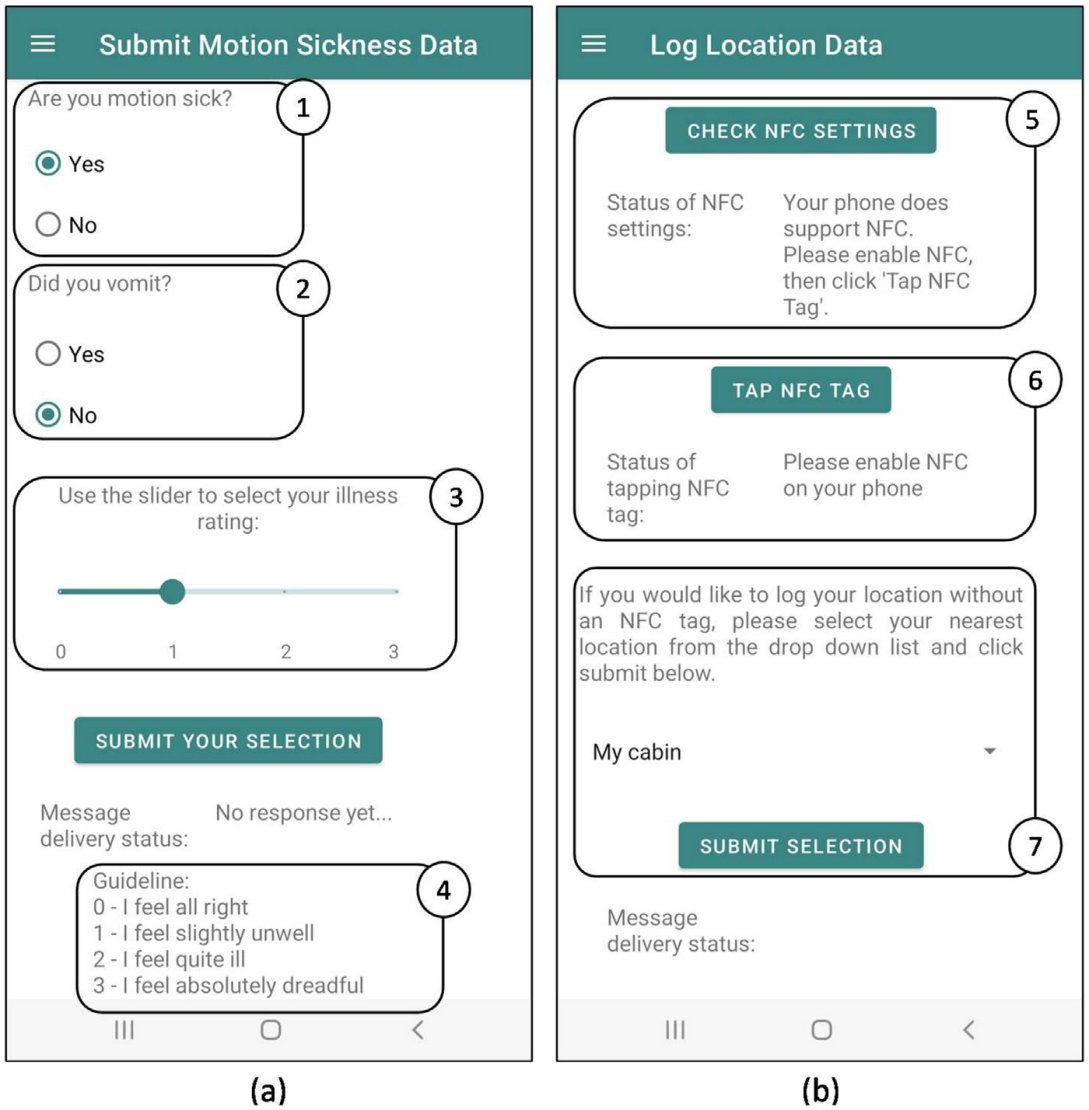
Mariner 4.0 application views for acquiring data from seafarers related to (a) motion sickness and (b) location [7].

The Mariner 4.0 application is a native Android mobile application developed in-house to facilitate mobile data collection from seafarers via their cell phones using the Wi-Fi network on the SA Agulhas II, South Africa’s polar supply and research vessel. The views for capturing motion sickness and location data are shown in Fig. 3.

In Fig. 3a, the individual-level subjective motion sickness incidence (MSI_subjective_), vomiting incidence (VI_subjective_) and illness rating (IR_subjectvie_) variables are captured from a seafarer with respective prompts: “Are you motion sick?” (1), “Did you vomit?” (2) and a slider to select their IR (3). A guideline for rating the severity of their motion sickness symptoms is provided (4), based on the original IR scale [8].

In Fig. 3b (5), the seafarer can inspect their device NFC functionality. If their smart phone is NFC-enabled, a seafarer can record their location using NFC tags (6). Otherwise, they can use a drop-down list (7) that corresponds to NFC tag locations. The locations of NFC tags distributed around passenger spaces on the SA Agulhas II are illustrated in Taylor *et al.* [1].

Notifications to remind seafarers to submit data through the Mariner 4.0 application are set to trigger three times a day at ship mealtimes, which was selected as a time when seafarers are expected to be by their cell phones. When a seafarer submits data, the time of submission and content (including their allocated Participant ID) are captured and sent to the HCPS via the Wi-Fi network.

### 4.2 Human response acquisition method 2 – paperbound questionnaires

Paper-based questionnaires, called daily diaries, were developed in earlier studies of human factors on the SA Agulhas II [9,10]. Each daily diary briefs voluntary participants on descriptions of relevant terms, such as symptoms of motion sickness, and guidelines for completing the booklet. The same format was used to compile daily diaries for Winter Cruise.

**Fig. 4:**
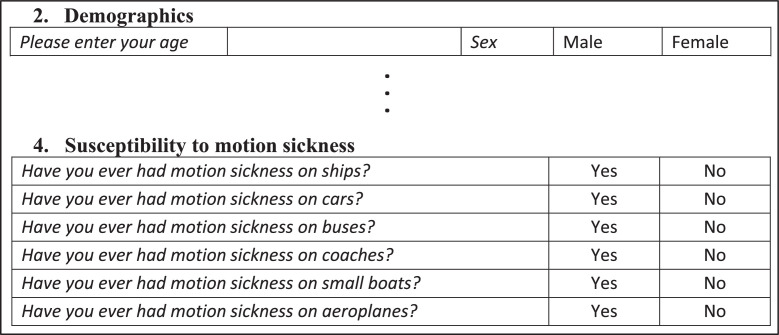
Motion sickness questions in the once-off questionnaire at the start of the daily diary (adapted [9,11]).

**Fig. 5:**
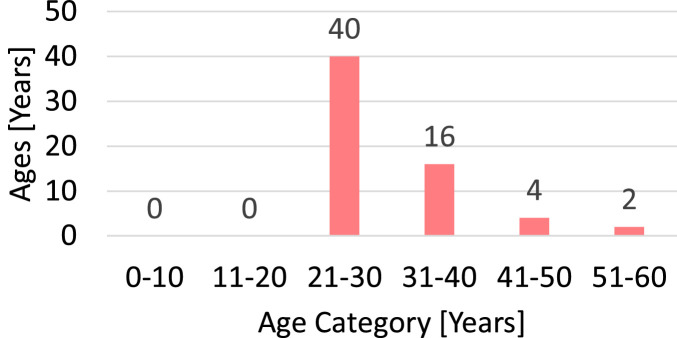
Age distribution of participants.

**Fig. 6:**
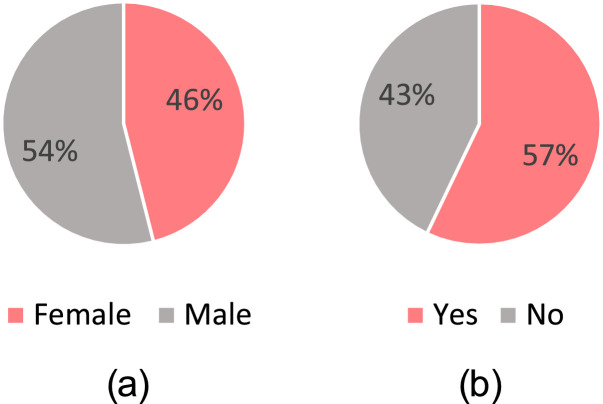
Demographics of participants (a) sex and (b) history of seasickness on ships.

The daily diary includes a once-off questionnaire at the start to record background and demographic information unique to the participant, as shown in Fig. 4 for questions related to motion sickness. The age distribution of participants is shown in Fig. 5 and the responses to sex and history of susceptibility to motion sickness on ships are shown in Fig. 6a and b, respectively.

**Fig. 7:**
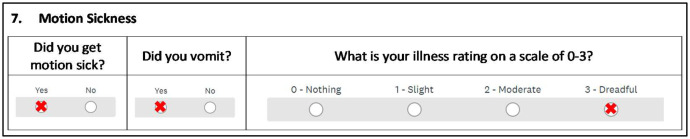
Motion sickness related questions in the daily questionnaire in the daily diary (adapted from [9]).

After the once-off questionnaire, a one-page questionnaire, as shown in Fig. 7 (showing only motion sickness questions), is repeated for each day of the voyage. The one-page questionnaire captures feedback from the participants regarding their daily perception and experience of motion sickness. Each question is related to a specific motion sickness metric, where:

• “Did you get motion sick?” is the MSI_subjective_;
• “Did you vomit?” is the VI_subjective_; and
• “What is your illness rating on a scale of 0-3?” is the IR_subjective_.

### 4.3 Ship response measurement

Direct Current (DC) accelerometer readings from a full-scale measurement system, described by Bekker *et al.* [3], were used for their measurement range that spans low frequencies of interest to motion sickness (below 1 Hz [6]). The accelerometers have been calibrated in a laboratory to verify measurement certainty. The accelerometers are positioned to form an array for translating z-oriented acceleration measurements to other points of interest on the ship. This method was developed to determine the rigid body motion of the SA Agulhas II using translational accelerometers [12].

**Fig. 8:**
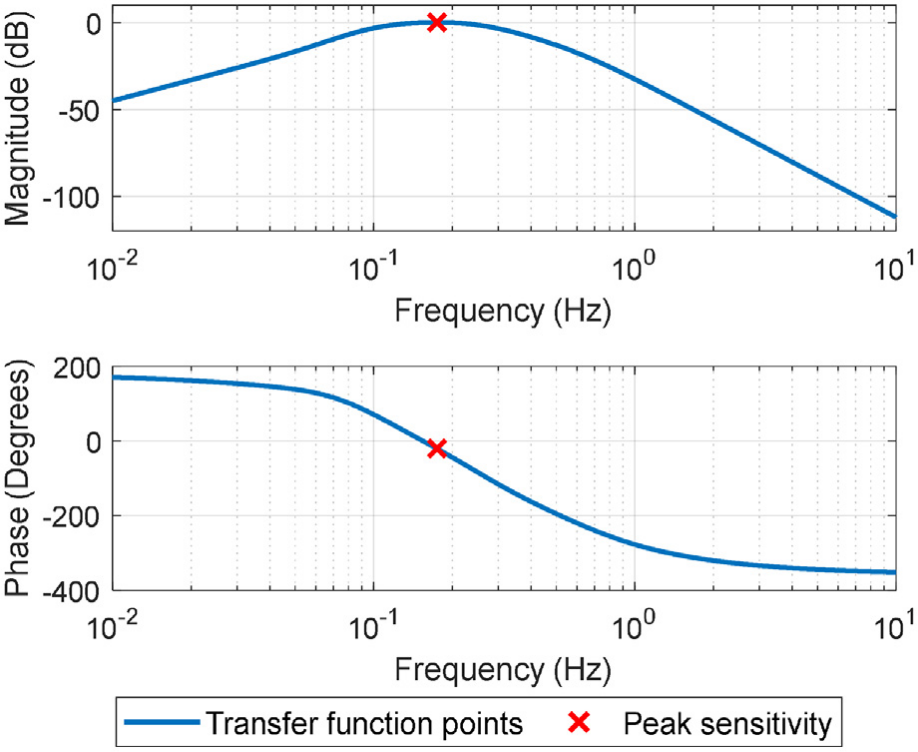
Bode plot for the transfer function of the *W*_f_ weighting filter (adapted from [6]).

A filter using a frequency weighting for motion sickness assessment is applied to the acceleration time signals using an infinite impulse response filter [[6], [13], [14]]. The total weighting filter for motion sickness, *W*_f_, is described by Eq. (1),

(1) $$W_{f}=H_{h}\left(s\right)\cdot H_{l}\left(s\right)\cdot H_{t}\left(s\right)\cdot H_{s}(s),$$

as provided in [6]. A high-pass filter, $H_{h}$, and low-pass filter, $H_{l}$, form a bandpass filter that preserves data within the frequency range of 0.08 Hz and 0.63 Hz. The combination of acceleration-velocity transition, $H_{t}$, and upward step, $H_{s}$, filters manipulates the measured signal to analytically represent human vibration perception. A Bode plot for the transfer function of the *W*_f_ weighting filter is presented in Fig. 8. Signal content between 0.1 and 0.5 Hz is preserved, with a peak sensitivity around 0.175 Hz, which allows for assessment of acceleration that is known to induce motion sickness [6].

### 4.4 MSDV computation

An MSDV is computed for each 5-minute file stored by the full-scale measurement system at all NFC tag and generic motion measurement locations. An MSDV_5min_ is computed using Eq. (2),

(2) $$\begin{array}{c} \text{MSD}V_{5\text{min}}={\left(T\right)}^{1/2}\cdot {\left(\frac{1}{N}\cdot \sum_{n=1}^{N}a_{w}^{2}\left(n\right)\right)}^{1/2}, \end{array}$$

where *T* is the duration of measurement (5 minutes), *N* is the number of samples and *a*_w_ is the weighted z-oriented acceleration [6].

### 4.5 Participant recruitment and briefing

**Fig. 9:**
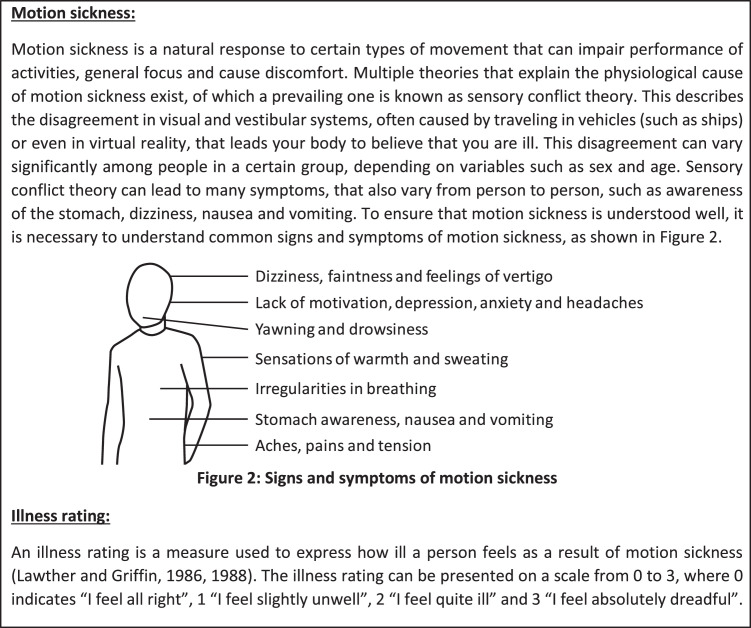
Definition section related to motion sickness in a daily diary (adapted from [9]).

All passengers (83) were invited to a recruitment presentation before departure for Winter Cruise on the evening of 11 July 2023. The presentation was performed in the Auditorium of the SA Agulhas II where participation materials were distributed before passengers arrived, including the daily diaries and written consent forms. Participation in research activities was detailed, making use of descriptions in the daily diaries. The definitions of motion sickness, its accompanying symptoms and the illness ratings that were used are shown in Fig. 9. Participants were informed that a “Yes” for “Are you motion sick?” should correspond to an IR of greater than 0, and a “No” with an IR of 0, otherwise the data interpretation conflicts.

Participant data collection through the Mariner 4.0 application and daily diaries were addressed. Fifteen (15) passengers volunteered to participate in providing human responses via both methods of subjective data acquisition, where 63 passengers participated in completing daily diaries. Participants were encouraged to:

• log submissions through the Mariner 4.0 application **at least three times a day** during mealtimes (i.e., breakfast at 07h30-08h30, lunch at 11h30-12h30 and dinner at 18h30-20h00 ship time). Ship time was kept to UTC+2hr during Winter Cruise, where UTC is coordinated universal time, but data submitted was recorded on their mobile devices in UTC+0h epoch time.
• complete their daily questionnaires **each morning**, reflecting on the previous 24 hours (from 08h00 to 08h00 ship time) when answering their questions.

## Limitations

The number of participants that contributed to this dataset does not allow for general inference of results beyond that of this study due to the sample size. However, the sample of passengers that participated in the traditional method of collecting human responses is suitable for making inferences for the population on board the SA Agulhas II during the Winter Research Voyage. Further data acquisition is required to make general inferences using a larger sample pool.

Fewer passengers participated in data collection through the HCPS. The limited participation is suspected to be due to the platform-specific mobile application (only Android) and possible undesired participation in parallel subjective data acquisition methods. Nonetheless, the HCPS enabled a higher frequency of human responses, such that the confidence in human responses for individuals is higher than that for the traditional paper-based method.

Human responses in this dataset are subjective and could be influenced by human factors outside of the scope of consideration in this study. Feedback pertaining to participant demographics, motion sickness incidence, vomiting incidence and severity of motion sickness symptoms were focussed on in this study exclusively.

The acceleration measurements of ship responses included in this dataset is a down-sampled subset of the greater dataset collected during the Winter Research Voyage. The greater dataset is too large to include in the online repository and can be accessed upon reasonable request to the authors.

## Ethics statement

Written consent was obtained from participants before participation during a recruitment and briefing session. The research was conducted under approval of the Research Ethics Committee: Social, Behavioural and Education Research of Stellenbosch University with ID: ING-2021-19463 and received institutional permission with ID: RPSD-2082. The study conducted to generate the dataset followed the required guidelines for ethical research practices [12].

## CRediT author statement

**Nicole Catherine Taylor:** conceptualisation, methodology, software, validation, investigation, data curation, writing – original draft, **Anriëtte Bekker:** conceptualisation, methodology, validation, supervision, resources, writing – review and editing, project administration, funding acquisition, **Karel Kruger:** conceptualisation, methodology, validation, supervision, resources, writing – review and editing, project administration.

## Data Availability

SUNScholarDataShip Motion Measurements and Human Responses Captured on the SA Agulhas II - Winter Cruise 2022 (Original data).
